# Near-Infrared Spectroscopy for Spinal Cord Monitoring—A Roadmap to Translational Research in Aortic Medicine

**DOI:** 10.1055/s-0043-1772774

**Published:** 2023-11-10

**Authors:** Konstantin von Aspern, Josephina Haunschild, Jens Garbade, Christian D. Etz

**Affiliations:** 1 Department for Cardiothoracic Surgery, Klinikum Links der Weser, Bremen, Germany; 2 Leipzig Heart Center, University Department for Cardiac Surgery, Leipzig, Saxony, Germany

**Keywords:** collateral network near-infrared spectroscopy, cnNIRS, spinal cord injury, spinal cord monitoring, translational research

## Abstract

Extensive aortic aneurysms represent a unique challenge necessitating interdisciplinary efforts for safe and effective treatment. Despite various adjunctive neuroprotective strategies, ischemic spinal cord injury remains a devastating complication. This article describes the implementation of collateral network near-infrared spectroscopy as the first noninvasive spinal cord monitoring modality in the setting of extensive open and endovascular aortic repair, from early conceptualization to clinical utilization. Potential capabilities and remaining uncertainties based on current evidence are outlined and discussed.

## Introduction

Treatment of extensive thoracoabdominal aneurysm disease via any modality represents a unique challenge. Despite various adjunctive neuroprotective strategies during and after open and endovascular aortic repair, ischemic spinal cord injury (SCI) remains a common and devastating complication with profound impact on individual long-term outcome, subsequent health care costs, and quality of life. 1 Specifically, for Crawford type II aneurysm repair, the incidence of postoperative neurologic deficits is reported at approximately 10 to 20%, based on clinical series and meta-analyses. 2 3 4

Since the first successful thoracoabdominal aortic operations in the early 1950s, 5 specialists in the field of aortic medicine have sought to overcome this problem.

Maintenance of adequate spinal cord tissue oxygenation is critical to prevent SCI during periods of acutely impaired blood flow.

Reliable monitoring of spinal cord integrity is required to allow for rapid response via hemodynamic, cardiopulmonary, and cerebrospinal fluid management.

Although some invasive tools for monitoring the spinal cord (e.g. motor-evoked potentials [MEP] and somatosensory-evoked potentials [SSEP]) are widely accepted, to date no validated method for noninvasive real-time monitoring has made its way into widespread clinical practice. This article describes the systematic translational research approach to a novel, noninvasive spinal cord monitoring modality using near-infrared spectroscopy.

### Current Spinal Cord Perfusion Concept—The “Collateral Network”

**Fig. 1:**
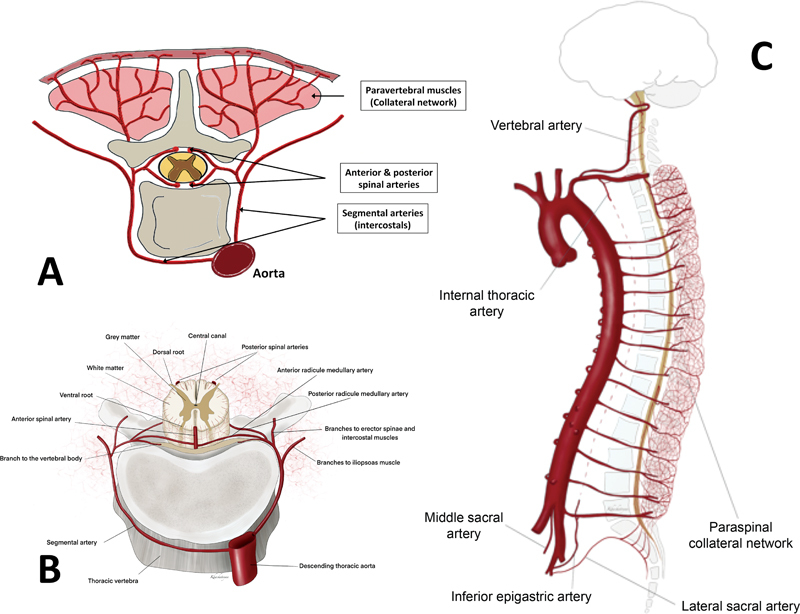
The paraspinal collateral network (CN)—( **A** ) schematic illustration of the CN (collateral network; image modified from von Aspern et al 1 ), ( **B** ) illustration of spinal cord blood supply from the dorsal segmental artery feeders (image modified from Khachatryan et al 8 ), ( **C** ) illustration of the paraspinal CN in relation to the upper thoracic and lower lumbar inflow (image modified from Khachatryan et al 8 ).

Comprehensive translational research over the past two decades has led to a better understanding of the dynamic arterial network ensuring the integrity of spinal cord perfusion, progressively challenging the historic paradigm and suggesting new strategies for spinal cord protection during and after thoracoabdominal aortic aneurysm (TAAA) repair. 6 Referred to as the “collateral network” (CN), this dynamic arterial network is largely localized in the paraspinal musculature and is fed by branches of the subclavian arteries, hypogastric artery, and directly from aortic segmental arteries (SAs 1 3 7 8 ; Fig. 1 ).

The CN is augmented by intraspinal—immediately available—circulatory circuits composed of repetitive, “Willis-like” micronetworks. 1 9 Through changes in regional blood pressure—caused by inflow disruption (e.g., SA occlusion)—the plasticity of the CN is capable of developing a robust alternative blood supply by means of arteriogenesis of new, and caliber alterations of preformed, arteries and arterioles. 1 10 This “priming” process is triggered after partial and complete aortic SA sacrifice, similar to open surgical and endovascular TAAA repair, 1 with the consequence of enabling sufficient blood flow to the spinal cord tissue during chronic or acute perfusion loss. 1 3 7

### Conventional Spinal Cord Monitoring

In order to circumvent imminent damage to the spinal cord tissue (intra- and postoperatively), few monitoring modalities have been developed or become routinely used in clinical practice. The most widely used invasive methods for intraoperative spinal cord monitoring are MEP and SSEP measurements. 11 Although SSEP monitoring can be implemented during the postoperative period, MEP monitoring on the awake, nonsedated patient is limited. 12 13 Usually, significant technical and human resources are required. These invasive methods are oftentimes not readily applicable during the postoperative course, while entailing various additional limitations (e.g., sedation and induced muscle relaxation). Ideally, spinal cord monitoring should reflect perfusion and ultimately tissue oxygenation in real time, so as to allow for rapid response in hemodynamic, cardiopulmonary (e.g. atrial fibrillation, ventilation), and cerebrospinal fluid management.

### Noninvasive Spinal Cord Monitoring: Collateral Network Near-Infrared Spectroscopy

Maintenance of adequate spinal cord oxygenation is critical to prevent SCI during periods of acutely impaired blood flow. Although invasive tools for monitoring the spinal cord (e.g. MEP and SSEP) are widely accepted, 14 for a long time no validated method for noninvasive real-time monitoring has made its way into clinical routine. Near-infrared spectroscopy utilizes characteristic adsorption spectra of oxygenated and deoxygenated hemoglobin at near-infrared wavelengths (760–2,500 nm) to quantify the regional tissue oxygenation (StO _2_ ) and consecutively estimate local perfusion. 1 8

**Fig. 2:**
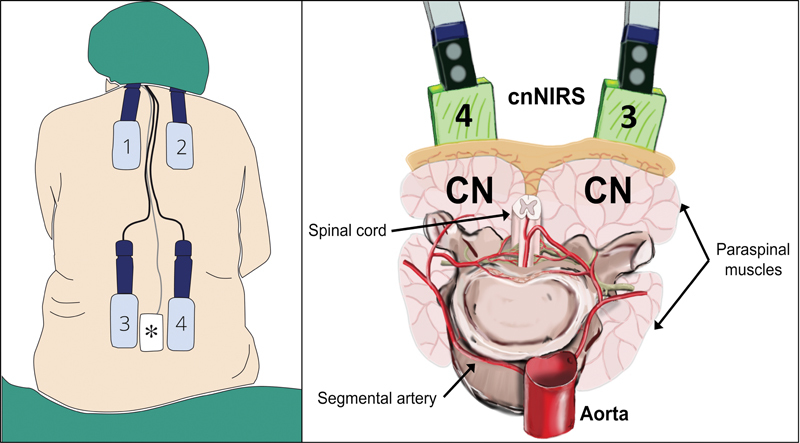
Near-infrared spectroscopy of the collateral network (cnNIRS). ( *Left* ) illustration of conventional bilateral optode placement pattern (thoracic and lumbar; image modified from Etz et al 26 ). ( *Asterisk* ) cerebrospinal fluid drainage; ( *Right* ) illustration of cnNIRS optodes with regard to the paraspinal collateral network (image modified from von Aspern et al 21 ). CN = collateral network.

Previous attempts to directly assess spinal cord tissue oxygenation using near-infrared spectroscopy have not been successful. The unfavorable bone-to-tissue ratio when placing the optodes directly above the spinous processes (axially in mid-line) limited direct oxygenation measurements of the spinal cord tissue, and measurements were clinically not useful. 1 4 15 16 Therefore, near-infrared spectroscopy of the collateral network (cnNIRS) has been introduced for noninvasive (indirect) real-time monitoring of spinal cord perfusion and oxygenation in TAAA repair 4 17 18 ( Fig. 2 ).

## The Translational Research Process

### Method Conceptualization and Validation

**Fig. 3:**
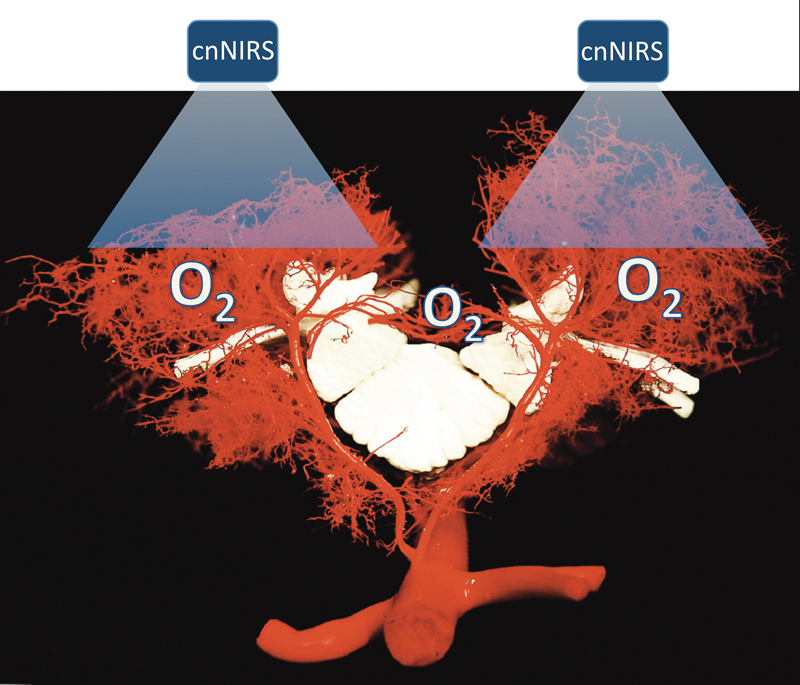
Image illustrating the rationale for noninvasive near-infrared spectroscopy monitoring of the collateral network (cnNIRS). The assumption is that oxygenation in the paraspinal collateral network compartment reflects spinal cord tissue oxygenation in real time. (Image modified from Bischoff et al 27 ).

Based on the CN concept, it was theorized that oxygenation and perfusion in the paraspinal muscles—hence the CN—should reflect oxygenation and perfusion of the spinal cord tissue ( Fig. 3 ). Therefore, two major assumptions needed to be confirmed.

1. Does oxygenation and perfusion in the paraspinal CN correlate with spinal cord tissue oxygenation and perfusion?
2. Does lumbar cnNIRS correlate with spinal cord tissue oxygenation and perfusion in real time?

To validate cnNIRS as a tool for monitoring CN-oxygenation and ultimately spinal cord integrity, it needed to be compared with direct oxygenation measurements of the paraspinal muscles and the spinal cord. For this purpose, an acute large animal model was designed comparing laser Doppler flowmetry (LDF)—an already validated method for invasive direct flow- and oxygenation measurements—and paraspinal cnNIRS during aortic blood flow alterations. 18 The experiment was carried out on seven juvenile pigs (German landrace; weight 41–48 kg). NIRS optodes for noninvasive CN monitoring were placed bilaterally at the thoracic (T5–T6) and lumbar (L2–L3) levels. For invasive, direct oxygenation and perfusion measurements, LDF probes were introduced (1) into the paravertebral muscles and (2) intrathecally at thoracic (T5–T7) and lumbar (L2–L4) levels under vision after exposure of the spinal column and the dura. A lateral thoracotomy via the seventh intercostal space was performed in order to expose the thoracic aorta for ischemia and reperfusion introduction. The experimental sequence was (1) baseline, (2) cross-clamping (ischemia), and (3) release (reperfusion). This sequence was repeated four times in each animal.

This experiment gave insight into the physiology of spinal cord perfusion and confirmed that the paraspinal CN vasculature has indeed a strong direct association with spinal cord microcirculation. It was demonstrated that regional paraspinal muscle oxygenation reflects spinal cord tissue oxygenation and that lumbar cnNIRS reproducibly depicts tissue oxygenation of the paraspinal vasculature during distal aortic ischemia and reperfusion ( *R* = 0.51–0.52, *p* < 0.001). Within 30 seconds, lumbar cnNIRS significantly decreased, reaching its nadir after 8 minutes of ischemia (69 ± 6 percent-of-baseline), returning to baseline values within 40 seconds during reperfusion. 18

### Clinical Application of Noninvasive cnNIRS Monitoring in Aortic Repair

Based on the results of the large animal validation experiments, cnNIRS in theory should provide valuable information on the oxygenation status of the CN and, thereby, indirectly, of the spinal cord also in a clinical scenario. Since near-infrared spectroscopy has already been implemented in clinical practice for other purposes, and because of its noninvasive characteristics, two further key questions needed to be answered in order to pave the way for clinical implementation as a new monitoring modality in aortic repair.

1. Is lumbar cnNIRS clinically feasible?
2. Are reduced lumbar cnNIRS measurements associated with postoperative neurologic deficits (paraplegia and paresis)?

In clinical practice, near-infrared spectroscopy has been used effectively only to monitor cerebral oxygen saturation during CPB and selective cerebral perfusion. 4 Analogous to this, conventional near-infrared spectroscopy optodes were used to monitor tissue oxygenation of the thoracic and lumbar paraspinal muscles—hence the paraspinal CN—to provide real-time, noninvasive spinal cord monitoring, potentially indicating pending spinal cord ischemia. This was the first clinical study on cnNIRS prior to, during, and after extensive open, endovascular, and hybrid TAAA repair. 4

The study included 20 patients (mean age: 66 ± 10 years). Fifteen patients had open thoracoabdominal aortic repair (Crawford II and III), three had thoracic endovascular aortic repair (TEVAR; Crawford I), and two had a hybrid repair (Crawford II). It was demonstrated that noninvasive cnNIRS monitoring in extensive TAAA repair is feasible. Lumbar cnNIRS directly responded to aortic cross-clamping, reaching minimum values after 11 ± 4 minutes (74 ± 13 percent-of-baseline). Patients suffering from postoperative paraplegia ( *N* = 3) demonstrated significantly lower lumbar cnNIRS values compared with patients who did not experience neurologic deficits ( *p* = 0.041). 4 These findings were later also confirmed by other researchers, who were able to further demonstrate a significant correlation between pathological MEP and low cnNIRS measurements in a clinical setting. Patients who exhibited intraoperative MEP ratios lower than 50% also had significantly lower cnNIRS values compared to patients with nonpathological MEP measurements ( *p* = 0.037). 17

Also, in clinical multicentre collaboration, cnNIRS has been investigated during the endovascular TAAA repair of 109 patients. In this study, Lewis and colleagues found that, in comparison with MEP and SSEP, cnNIRS was less sensitive for detecting potential SCI (33% NIRS; 100% MEP/SSEP); however, cnNIRS was more specific in that regard (99% NIRS; 10% MEP; 12% SSEP). 19

### Translational Feedback Concept—Addressing Clinical Key Questions Experimentally

Although previous clinical and experimental studies have gradually shown that cnNIRS was technically feasible and that lumbar measurements correlate with spinal cord perfusion and oxygenation during and after aortic cross-clamping and reperfusion, 4 18 cnNIRS monitoring during consecutive SA occlusion has not been evaluated. Since extensive open and endovascular aortic repair entails SA sacrifice and, in light of the recent introduction of minimally invasive staged SA coil- and plug embolization (MIS ^2^ ACE) for paraplegia prevention, 3 6 20 real-time spinal cord oxygenation monitoring became increasingly important. According to the translational feedback concept of addressing clinical key questions using established large animal models, subsequent acute and chronic experiments were needed to answer two additional questions:

1. Does lumbar cnNIRS react to consecutive SA sacrifice in real time (comparable to the effects of extensive open aortic replacement or endovascular stenting)?
2. Is lumbar cnNIRS significantly correlated with neurologic outcome in a controlled experimental model (confirming previous clinical results)?

Through these experiments, 3 21 it was demonstrated for the first time in a controlled, chronic large animal experiment that lumbar cnNIRS reacts to occlusion of SAs in real time and correlates with neurologic outcome. These experiments included 12 juvenile pigs with SA occlusion via open surgery and consecutive ligation and 18 juvenile pigs with SA occlusion via staged minimally invasive coil embolization. 3 21 Subjects from the open surgery experiment were further subdivided into a total occlusion ( *N* = 7) and a subtotal occlusion group (mimicking reimplantation of crucial SAs with patent T12/T13, *N* = 5). The minimally invasive occlusion experiment also differentiated between left- and right-sided SA occlusion with regard to cnNIRS measurements. In both experiments, pigs were monitored over 3 days after finalization. Clinical status and neurologic evaluation were assessed regularly at 6-hour intervals. Neurologic outcome was evaluated using a modified Tarlov scoring system. 3 21 In the open surgery experiment, all subjects from the subtotal occlusion group completely recovered, whereas 57% of the total occlusion group were paraplegic ( *N* = 4/7). In the minimally invasive coil embolization experiment, permanent paraplegia occurred in two (11%) and any kind of neurological deficit—temporary or permanent—in seven animals (39%).

After complete SA occlusion, cnNIRS decreased—analogous to the ischemia/reperfusion experiments—from 90 ± 4% to 58 ± 9% (32% of baseline, *p* < 0.008) with significant correlation to neurologic outcome (R = 0.7, *p* < 0.001). 3 This preliminary data supported cnNIRS as a valuable noninvasive tool for detecting imminent spinal cord ischemia during and after aortic procedures involving SA occlusion.

### Implications of cnNIRS for Novel Spinal Cord Protection Methods

MIS ^2^ ACE has been introduced for paraplegia prevention prior to extensive aortic repair. During this procedure, SAs are occluded via a coil or plug insertion into the proximal portion of the vessel, inducing CN priming for subsequent definitive aortic repair. Due to frequent technical difficulties in localizing certain SAs for coil insertion in an aneurysmatic aorta and the potential risk for MIS ^2^ ACE-associated SCI, additional efforts to facilitate a safe and effective MIS ^2^ ACE procedure seemed warranted. 22 Exact knowledge of the SA position and angle is paramount in order to avoid repetitive contrast application or aortic injury due to excessive catheter manipulation during MIS ^2^ ACE. Correct occlusion assessment represents another issue, since incomplete SA occlusion may render an MIS ^2^ ACE procedure ineffective, while excessive occlusion or distal embolization may result in iatrogenic SCI.

In order to further simplify the MIS ^2^ ACE procedure, an additional acute as well as a chronic large animal experiment were designed to answer the question:

1. Is cnNIRS capable of guiding the MIS ^2^ ACE procedure by reliably detecting occlusion of individual SAs, thereby potentially minimizing the amount of contrast agent, radiation exposure, and overall duration of an MIS ^2^ ACE procedure?

**Fig. 4:**
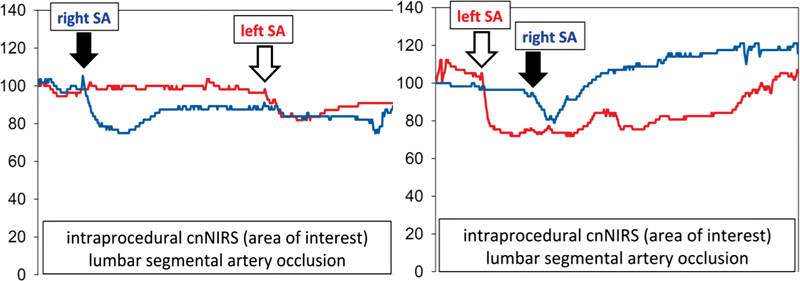
Intraprocedural real-time lumbar near-infrared spectroscopy of the collateral network (cnNIRS) measurements after occlusion of a left- ( *red lines* ) and right-sided ( *blue lines* ) lumbar segmental artery (SA), arrows indicate moment of separate SA coil embolization.

It was demonstrated for the first time that lumbar cnNIRS independently reacts to unilateral SA occlusion ( Fig. 4 ). The mean difference between left- and right-sided cnNIRS measurements was 7 ± 4% as soon as 1 minute after SAs of one side (and level) were occluded and perfusion via the contralateral side remained ( *p* = 0.001). 21 Lumbar cnNIRS also corresponded with neurologic outcome after MIS ^2^ ACE. 21 Based on these results, it was concluded that cnNIRS-guided SA occlusion is feasible and may provide a useful adjunct, facilitating adequate and complete vessel occlusion.

### Expanding and Optimizing Noninvasive Spinal Cord Monitoring

Previous studies have demonstrated the correlation of lumbar cnNIRS with the degree of spinal ischemia and neurologic outcome. 1 17 Furthermore, recent clinical and experimental studies have highlighted its efficacy for measuring the lumbar portion of the paraspinous CN (L2-L4) and demonstrated that high thoracic (T4-T6) cnNIRS measurements may be of limited clinical use (presumably due to the extensive collateralization in that region). 18 At the time no experience with cnNIRS measurements of the remaining paraspinal portion—hence the entire paraspinal CN—was available. Especially with regard to the plethora of procedures focused on the thoracic portion of the aorta, potentially contributing to a procedural-related SCI risk (such as aortic arch replacement with a frozen elephant trunk, fET), cnNIRS for real-time CN mapping needed to be investigated. Two main questions were addressed:

1. Does cnNIRS of the mid-thoracic region downwards (T7 to L5) react to distal aortic ischemia and reperfusion?
2. Is an expanded noninvasive cnNIRS optode placement pattern (T7 to L5) potentially a versatile monitoring method also for procedures limited to the proximal thoracic aorta (e.g. fET)?

The rational of these subsequent experiments was to compare segmental cnNIRS measurements to the corresponding direct CN and spinal cord regional perfusion (measured by microsphere injection) and thereby identify optimal cnNIRS optode placement pattern. It was reliably shown that cnNIRS is capable of detecting relevant changes during distal ischemia and reperfusion from the midthoracic level (T7) downward. 1 Measurements at the midthoracic to low lumbar levels decreased rapidly to a nadir at 10 minutes of distal ischemia (mean differences between 18 ± 11% and 44 ± 9% of baseline; *p* < 0.001–0.045), with more pronounced changes in the caudal regions. 1 High thoracic cnNIRS (T3–T6) remained stable, analogous to previous clinical and experimental studies. Measurements of cnNIRS, CN, and spinal cord regional perfusion demonstrated comparable curve progressions starting from the midthoracic region (R = 0.5–0.7; *p* < 0.001). 1 It was therefore concluded that for aortic procedures an expanded noninvasive cnNIRS optode placement (T7-L5) seems useful and may serve as a versatile monitoring method also for procedures limited to the proximal thoracic aorta. 1

In a direct consequence, various groups used cnNIRS clinically during extensive arch procedures utilizing the fET technique. 23 24 25 In these series, it was demonstrated that cnNIRS of the midthoracic region (starting from thoracic level 7) measures a significant decrease during fET implantation. 23 24 Honkanen and colleagues demonstrated in their large animal experiments that cnNIRS measurements at the mid- and lower thoracic region (T8–T10) significantly decrease during simulated fET implantation with selective cerebral perfusion, reaching a nadir within 35 minutes. 25 In a clinical series of 18 patients by Kinoshita et al *,* cnNIRS measurements at the thoracic level (T10) decreased markedly during circulatory arrest and fET implantation, even after initiation of selective cerebral perfusion (nadir at 30 percent-of-baseline after 20 minutes). Values increased and reached baseline values within 30 minutes after resuming full body circulation. 24 However, these experiments, clinical series, and reports were underpowered to demonstrate a clear correlation with neurologic outcome and remained speculative with regard to the potential clinical implications of this new monitoring modality in the setting of extended aortic arch procedures.

## Conclusion and Perspective

Despite various contemporary adjuncts to mitigate treatment-associated SCI, the incidence of paraplegia after open and endovascular TAAA repair remains high. Clinically established methods for spinal cord monitoring are invasive and oftentimes not readily applicable during the postoperative course. Ideally, spinal cord monitoring should be noninvasive and easy to use and should reflect tissue perfusion/oxygenation in real time.

This presented translational research review, aims at systematically investigating cnNIRS as a feasible monitoring method, from early conceptualization to clinical application in aortic medicine.

The outlined translational research approach is dynamic and ongoing. Based on the currently available data, however, lumbar cnNIRS reproducibly reflects spinal cord tissue oxygenation and perfusion in real time and appears a feasible method for clinical practice. cnNIRS reacts to open and endovascular SA sacrifice, functioning as a promising new tool for guiding spinal cord protective procedures such as MIS ^2^ ACE. Both experimentally and clinically, it has been demonstrated that cnNIRS correlates with postoperative neurologic outcome and other established monitoring modalities such as MEP and SSEP with high specificity. The data at hand indicate that an expanded optode placement pattern might be useful also for procedures limited to the proximal thoracic aorta (e.g. fET); however, further studies are warranted to generate adequate power for meaningful conclusions above and beyond current preliminary results.

As a consequence of a decade of systematic research, cnNIRS has since been introduced as a method for spinal cord monitoring during and after aortic repair at many specialized centers. Although additional clinical and experimental research is warranted (to further investigate cnNIRS with regard to different patient- and procedural-related aspects [e.g., hypothermia, SA reimplantation and sarcopenia], measurement thresholds indicative of imminent SCI and prolonged monitoring periods beyond 48 hours to account for potential delayed paraplegia), this noninvasive method has become a promising tool for spinal cord monitoring during and after aortic procedures of any modality.
